# Baicalin as a Regulator of Peroxisome Proliferator‐Activated Receptor Gamma: Alleviating Sepsis‐Induced Liver Injury by Inhibiting the Cluster of Differentiation 14/Nuclear Factor Kappa B Signaling Pathway

**DOI:** 10.1002/iid3.70319

**Published:** 2026-02-12

**Authors:** Hui Wang, Mengmeng Guo, Xueling Zeng, Yuqi Hu, Jiawei Ma, Yufei Chang

**Affiliations:** ^1^ Department of Emergency, Beijing Ditan Hospital Capital Medical University Beijing China

**Keywords:** baicalin, Cluster of Differentiation 14, liver injury, peroxisome proliferator‐activated receptor gamma, sepsis

## Abstract

**Background:**

Sepsis frequently results in multiple organ failure, with the liver particularly susceptible to sepsis‐induced damage. The flavonoid baicalin (BA), derived from *Scutellaria baicalensis*, presents anti‐inflammatory properties, yet its effects on liver injury in sepsis through the Cluster of Differentiation 14 (CD14)/Nuclear Factor kappa B (NF‐κB) pathway remain underexplored.

**Methods:**

Alpha Mouse Liver 12 (AML12) cells were intervened with different concentrations of BA and subsequently challenged with lipopolysaccharide (LPS) to induce inflammation. Cell viability, apoptosis, inflammatory cytokine production, and signaling pathway activation were tested by Cell Counting Kit 8 (CCK8), flow cytometry, quantitative real‐time PCR (qRT‐PCR) and western blotting, respectively. And immunofluorescence was employed to visualize nuclear ectopic location of NF‐κB in cells. Additionally, the impact of peroxisome proliferator‐activated receptor gamma (PPARγ) silencing for BA's function was explored using small interfering RNA (siRNA) techniques.

**Results:**

BA significantly improved viability and decreased apoptosis in LPS‐treated cells. It also downregulated tumor necrosis factor alpha (TNF‐α), interleukin‐1 beta (IL‐1β) and IL‐6, upregulated PPARγ, and diminished CD14 and phosphorylated inhibitor of kappa B alpha (p‐IKBα)/IKBα levels. Silencing PPARγ reversed the protective effects of BA, underscoring the importance of PPARγ in BA's mechanism of action.

**Conclusion:**

BA alleviates sepsis‐induced liver injury through activating PPARγ and inhibiting CD14/NF‐κB pathway, highlighting its potential as a new approach to treatment sepsis.

## Graphical abstract

Baicalin (BA) alleviates sepsis‐induced liver injury through activating peroxisome proliferator‐activated receptor gamma (PPARγ) and inhibiting the Cluster of Differentiation 14 (CD14)/Nuclear Factor kappa B (NF‐κB) pathway.

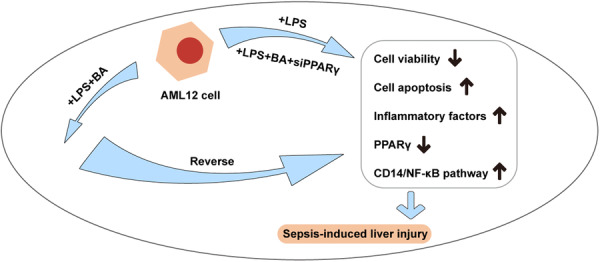

## 1 Introduction

Sepsis stands as a paramount concern in critical care settings, notorious for its alarmingly high mortality rates which are primarily attributed to its ability to induce multiple organ failure [1, 2]. The pathological process of sepsis involves an early systemic inflammatory overreaction and a subsequent prolonged stage of persistent immunosuppression, accompanied by a large number of immune cell apoptosis and immune dysfunction, resulting in a significant decline in the host's anti‐infection ability [3, 4]. This overwhelming infection‐induced systemic inflammatory can cause a rapid decline in physiological functions across multiple organ systems [2, 5, 6]. At the level of the immune mechanism, sepsis activates innate and adaptive immune responses through pathogen‐associated molecular patterns and damage‐associated molecular patterns, triggering cytokine storms, which subsequently cause tissue damage and organ dysfunction [3]. Among these, the liver is exceptionally vulnerable to sepsis‐induced damage [7]. As a vital organ in both metabolic processes and immune system regulation, the liver's impairment during sepsis is often a prognostic marker of severe complications and can dictate the clinical outcomes of septic episodes [8].

The vulnerability of the liver in septic conditions can be attributed to its central role in filtering bacteria and toxins from the blood, producing crucial proteins for clotting and immune function, and regulating a vast array of metabolic functions that are essential for homeostasis [9]. During sepsis, the liver is subjected to a cascade of cytokines and inflammatory mediators that can disrupt its function, which can result in hepatocellular injury, disrupted protein synthesis, and impaired bile production, all of which exacerbate the systemic response to infection [7, 9].

Given the critical role of the liver in managing infectious agents and maintaining metabolic equilibrium, strategies focused on preserving or restoring liver function are of utmost importance [10, 11]. Such strategies not only aim to stabilize key hepatic functions but also mitigate the systemic inflammatory responses typically observed in septic shock. Improving liver function can significantly enhance the prognostic outlook for septic patients, potentially reducing morbidity and mortality associated with this condition. At present, there are no specific drugs for liver damage caused by sepsis in clinical practice, and the treatment strategies mainly focus on comprehensive management of sepsis and liver protection therapy. Commonly used medications include anti‐infective drugs, liver‐protective drugs, and immunomodulators, such as Meropenem, polyene phosphatidylcholine, hydrocortisone, and so forth. Although these drugs have certain anti‐inflammatory, detoxifying, or liver‐protecting effects, they also have some side effects: glucocorticoids have a strong immunosuppressive effect and may increase the risk of secondary infections. Therefore, it is of great significance to find alternative drugs with multiple targets, high efficiency and good safety, and exploring novel therapeutic agents that can directly modulate key inflammatory mediators becomes crucial.

The toll‐like receptor 4 (TLR4) and its high‐affinity ligand receptor complex consisting of lipopolysaccharide (LPS) and LPS‐binding protein (LBP) play pivotal roles in the immune response to bacterial infections [12, 13, 14, 15]. Cluster of Differentiation 14 (CD14), a co‐receptor for LPS–LBP, is expressed on various cell membranes and is instrumental in mediating this response, facilitating the activation of TLR4 and subsequent propagation of the pro‐inflammatory cascade [16, 17, 18, 19]. In liver tissue, CD14 contributes to injury progression, primarily through the modulation of inflammatory pathways that can exacerbate tissue damage during sepsis [20, 21, 22, 23].

Baicalin (BA), a flavonoid derived from the dried roots of *Scutellaria baicalensis* (*S. baicalensis*), exhibits multiple biological activities, such as suppressing inflammation and cancer [24, 25, 26]. While its role in sepsis has been explored, research focusing specifically on BA's effects on sepsis‐associated liver injury remains sparse [24]. Previous studies have demonstrated that BA can stimulate nuclear receptor peroxisome proliferator‐activated receptor gamma (PPARγ), a known negative regulator of CD14, thereby potentially modulating the inflammatory response driven by the nuclear factor kappa B (NF‐κB) pathway in septic conditions [27, 28, 29].

Given the complexity of sepsis and the central role of the liver in mediating systemic responses, understanding the potential of BA to modulate key inflammatory pathways through mechanisms such as PPARγ activation could open new therapeutic avenues. This research aims to delineate the potential of BA in treating sepsis‐induced liver injury through its interactions with CD14 and the downstream NF‐κB signaling pathway in a cellular model of sepsis.

## 2 Materials and Methods

### 2.1 Cell Culture

Bioresource Collection and Research Center (60326, China) provided Alpha Mouse Liver 12 (AML12) cells, and mycoplasma contamination and Short Tandem Repeat analysis were checked. Dulbecco's Modified Eagle Medium (DMEM) (C0891, Beyotime, China) with 10% fetal bovine serum (C0235, Beyotime, China) and 1% penicillin–streptomycin (CA005‐010, GenDEPOT, USA) was administered to culture cells at 37°C in a humidified incubator (95% air/5% CO_2_) until they reached 70%–80% confluency.

### 2.2 Small Interfering RNA (siRNA) Transfection

Gene silencing was performed by transfecting AML12 cells with synthetic siRNA using Lipofectamine 2000 (BL623A, Biosharp, China). Cells were transfected with 50 nM of either PPARγ ‐ targeting siRNA (siPPARγ; sense sequence: 5′‐GCUAUGACCAGCUAGCUAU‐3′) or a nontargeting control siRNA (siNC) according to the manufacturer's instructions. After transfection, cells were incubated for 48 h (37°C), followed by collection and further examination. Transfection efficiency was verified using quantitative real‐time PCR (qRT‐PCR).

### 2.3 Group Processing

To assess cell viability, AML12 cells were divided into five groups: 0, 25, 50, 100, and 150 groups, where cells were treated with 0, 25, 50, 100, or 150 µM BA (572667, Sigma‐Aldrich, USA), respectively, for 30 min [30]. Subsequently, 25 and 100 µM BA concentrations were selected to continue the cell viability assessment. The cells were assigned into four groups: Control group (normal culture); LPS group (24‐h treatment with 0.5 µg/mL LPS (ST1470, Beyotime, China) to induce an inflammatory response) [31]; LPS + BA (low) group (30‐min treatment with 25 µM BA followed by LPS treatment); LPS + BA (high) group (30‐min treatment with 100 µM BA followed by LPS treatment). To examine the impact of PPARγ silencing on BA's mitigation of sepsis, the AML12 cells were distributed into LPS group, LPS + BA group (30‐min treatment with 100 µM BA followed by 0.5 µg/mL LPS for 24 h), LPS + BA + siNC group (transfection with siNC and 30‐min treatment with 100 µM BA followed by LPS treatment), and LPS + BA + siPPARγ group (transfection with siPPARγ and 30‐min treatment with 100 µM BA followed by 0.5 µg/mL LPS for 24 h).

### 2.4 Cell Counting Kit 8 (CCK8) Assay

CCK8 (ab228554, Abcam, UK) was used to assess cell viability. In short, AML12 cells were seeded in 96‐well plates (FPT011, Beyotime, China) and treated as described. At 24, 48, and 72 h posttreatment, added 10 µL CCK8 solution, and incubated (2 h, 37°C). The viability was assessed by measuring the absorbance at 460 nm using a microplate reader (Tecan Infinite M200 PRO, Tecan, Switzerland). The results were expressed as a percentage of relative to the control (0 µM BA).

### 2.5 Flow Cytometry for Apoptosis

A Cell Apoptosis Kit (640914, BioLegend, USA) was used following the kit's instruction. Following 48‐h treatment, cells were collected, washed with cold phosphate‐buffered saline (C0221A, Beyotime, China), and re‐dispersed in 195 µL binding buffer. In total, 5 µL Annexin V‐FITC and 10 µL propidium iodide were added to incubate cells (15 min, darkness, room temperature), and then 400 µL binding buffer was added to each sample. Apoptosis was quantified using a NovoCyte Penteon flow cytometer (Agilent, USA).

### 2.6 QRT‐PCR

Total RNA from AML12 cells was isolated using TRIzol (R0016, Beyotime, China), and transcribed into cDNA utilizing SuperScript IV First‐Strand Synthesis System (18091050, Thermo Fisher Scientific, USA). The reaction parameters were set as follows: 12 min of 25°C, 2 h of 37°C, and 10 min of 90°C to deactivate the reverse transcriptase. QRT‐PCR was conducted with the Mix (K0253, Thermo Fisher Scientific, USA) on a System (Applied Biosystems, USA). Each 10 µL reaction solution contained 5 µL Mix, 0.5 µL primer (10 µM, Tsingke Biotechnology, China), 1 µL cDNA, and 3 µL nuclease‐free water. The following primer sequences were used: tumor necrosis factor alpha (TNF‐α): 5′‐CCTCTCTCTAATCAGCCCTCTG‐3′ (F), 5′‐GAGGACCTGGGAGTAGATGAG‐3′ (R); interleukin‐1 beta (IL‐1β): 5′‐GAAATGCCACCTTTTGACAGTG‐3′ (F), 5′‐TGGATGCTCTCATCAGGACAG‐3′ (R); IL‐6: 5′‐TAGTCCTTCCTACCCCAATTTCC‐3′ (F), 5′‐TTGGTCCTTAGCCACTCCTTC‐3′ (R); PPARγ: 5′‐GGAAGACCACTCGCATTCCTT‐3′ (F), 5′‐GCGGTCTCCACTGAGAATAATG‐3′ (R); Glyceraldehyde 3‐Phosphate Dehydrogenase (GAPDH): 5′‐AAGGTCATCCCAGAGCTGAA‐3′ (F), 5′‐CTGCTTCACCACCTTCTTGA‐3′ (R). Thermal cycling parameters were set as follows: first denaturation (90°C, 15 min), 35 cycles (90°C for15 s, 65°C for 30 s, and 68°C for 30 s). At last, a melting curve analysis was used, and the related mRNA levels were quantified by the 2^−ΔΔCt^ method [32].

### 2.7 Western Blot

Based on previous studies [33], the levels of related proteins were detected using Western Blot. In short, RIPA buffer (P0013, Beyotime, China) was used for cell lysis. Total protein was separated by centrifugation (12,000 rpm, 20 min, 4°C) and was quantified by a Protein Assay Kit (08W00021, MP Biomedicals, USA). Samples (30 μg) were separated by sodium dodecyl sulfate polyacrylamide gel electrophoresis (C671104, Sangon Biotech, China) and transferred onto Polyvinylidene Fluoride membranes (F619537‐0001, Sangon Biotech, China). Membranes were blocked by 5% skimmed milk (1 h), probed with primary antibodies (PPARγ (58 kDa, ab209350, Abcam, UK), CD14 (50 kDa, ab221678, Abcam, UK), phosphorylated Inhibitor of kappa B alpha (p‐IKBα) (40 kDa, ab133462, Abcam, UK), IKBα (35 kDa, ab32518, Abcam, UK), and GAPDH (36 kDa, ab128915, Abcam, UK)) overnight at 4°C, and then treated with secondary antibody (ab205718, Abcam, UK) (24°C, 1 h). ECL system (Amersham Pharmacia, Piscataway, NJ, USA) was exploited for chemiluminescence detection.

### 2.8 Immunofluorescence

The NF‐κB activation nuclear translocation was detected by immunofluorescence [34]. AML12 cells were fixed (24 h) with 10% buffered formalin (E672001‐0001, Sangon Biotech, China), washed and then cultured with 0.1% Triton X‐100 (ST1723, Beyotime, China) (15 min) to permeabilize cell membranes. Cells were blocked with 5% bovine serum albumin (A610903, Sangon Biotech, China) (1 h, room temperature) and incubated (overnight, 4°C) with primary anti‐NF‐κB antibody (ab207297, Abcam, UK). Cells were washed and incubated (37°C, 1 h) with secondary antibody (ab150080, Abcam, UK). 4′,6‐diamidino‐2‐phenylindole (DAPI) (C1002, Beyotime, China) was used for nuclei stain, and a BX53 microscope (Olympus Life Science, Japan; 200× magnification; scale = 50 μm) was used for imaging. Blue represents cell nuclei stained with DAPI and red represents NF‐κB. For quantitative analysis, three randomly selected fields per group sample were used.

### 2.9 Statistical Analysis

**Figure 1:**
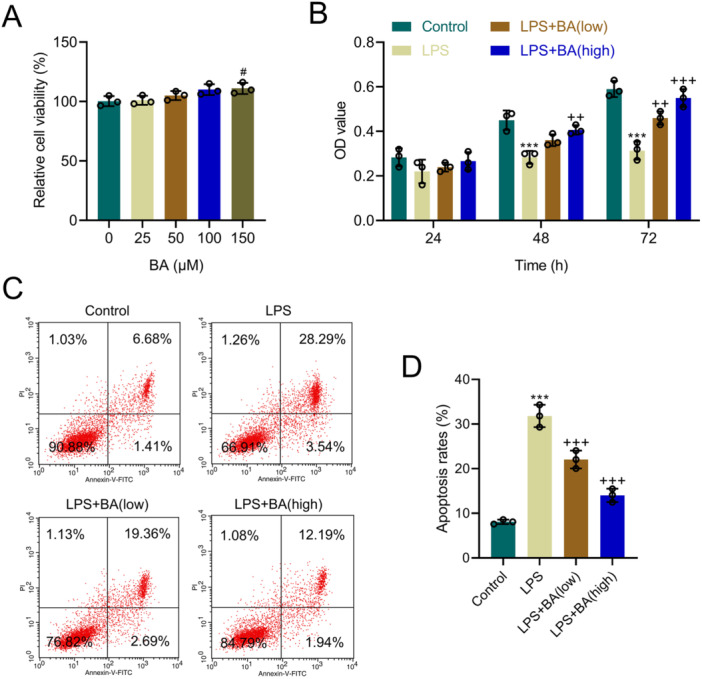
Effects of baicalin (BA) on Alpha Mouse Liver 12 (AML12) cell viability and apoptosis induced by lipopolysaccharide (LPS). (A) Cell viability of AML12 cells from the 0, 25, 50, 100, and 150 groups, as assessed by the Cell Counting Kit 8 (CCK8) assay. (B) Cell viability of AML12 cells from the Control, LPS, LPS + BA (low), and LPS + BA (high) groups for 24, 48, and 72 h. (C and D) Flow cytometry analysis of apoptosis rates in AML12 cells from the Control, LPS, LPS + BA (low), and LPS + BA (high) groups for 48 h. *n* = 3. ^#^ versus 0, *p* < 0.05; *** versus Control, *p* < 0.001; ^++^versus LPS, *p* < 0.01, ^+++^ *p* < 0.001.

Data analysis was conducted using Prism 8.0 software (GraphPad Software, USA). One‐way ANOVA was used to perform the multigroup comparisons. For Figure 1A, Dunnett's post hoc test was used for multiple comparisons between groups, while Tukey's post hoc test was for the rest of comparisons to determine significant differences between groups. Statistical significance was deemed at *p* < 0.05. Results are presented as mean ± standard deviation.

## 3 Results

### 3.1 Effect of BA on LPS‐Induced Viability and Apoptosis of AML12 Cells

Using the CCK8 assay, how BA impacted AML12 cells viability was examined. The results indicated that concentrations of 0/25/50/100 µM BA had no impact on cell viability, whereas 150 µM BA enhanced cell viability (*p* < 0.05, Figure 1A). Thus, we selected nonimpactful concentrations of BA (25 and 100 µM) for subsequent studies. Further viability assays indicated that the LPS group had reduced OD values at 48 and 72 h (*p* < 0.001, Figure 1B); compared to the LPS group, low‐concentration BA improved the OD values at 72 h, and high‐concentration BA improved OD values at both 48 and 72 h (*p* < 0.01, Figure 1B). This indicates that BA can mitigate LPS‐induced suppression of AML12 cell viability. Flow cytometry results revealed the apoptosis rate of AML12 cells at 48 h in LPS group was increased, while BA could alleviate LPS‐induced apoptosis (*p* < 0.001, Figure 1C,D).

### 3.2 Regulation of TNF‐α, IL‐1β, and IL‐6 and CD14/NF‐κB Pathway by BA

**Figure 2:**
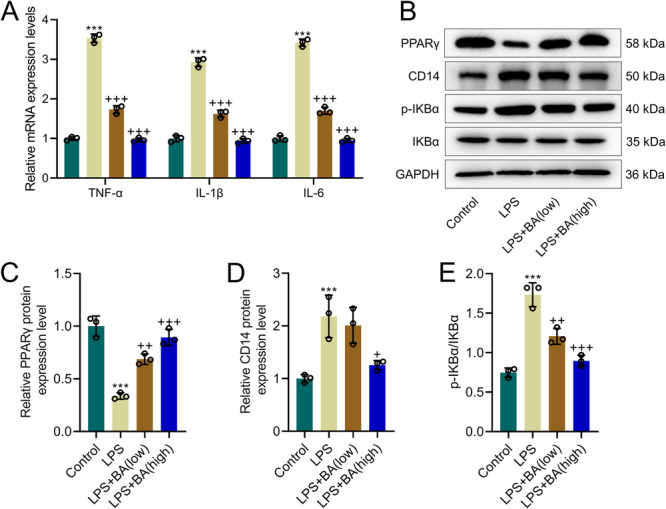
BA mitigates LPS‐induced inflammatory responses and modulates peroxisome proliferator‐activated receptor gamma (PPARγ), Cluster of Differentiation 14 (CD14), and nuclear factor kappa B (NF‐κB) pathways in AML12 cells. (A) Quantitative real‐time PCR (qRT‐PCR) analysis of relative mRNA expression levels of tumor necrosis factor alpha (TNF‐α), interleukin‐1 beta (IL‐1β), and IL‐6 from the Control, LPS, LPS + BA (low), and LPS + BA (high) groups, with Glyceraldehyde 3‐Phosphate Dehydrogenase (GAPDH) as a loading control. (B–E) Western blot analysis of PPARγ, CD14, phosphorylated Inhibitor of kappa B alpha (p‐IKBα), and IKBα protein levels from the Control, LPS, LPS + BA (low), and LPS + BA (high) groups, with GAPDH as a loading control. *n* = 3. *** versus Control, *p* < 0.001; ^+^ versus LPS, *p* < 0.05, ^++^ *p* < 0.01, ^+++^ *p* < 0.001.

QRT‐PCR results showed TNF‐α, IL‐1β, and IL‐6 levels were increased in the LPS group compared to the Control group, while BA was able to mitigate the elevation of these inflammatory markers induced by LPS (*p* < 0.001, Figure 2A). Western blot indicated PPARγ protein levels were decreased in LPS group, while levels of CD14 and p‐IKBα/IKBα were increased. High concentration of BA was able to reverse the effects of LPS on the expression of these proteins, and low concentration of BA could reverse the impact of LPS on the expression of PPARγ and p‐IKBα/IKBα (*p* < 0.05, Figure 2B–E), indicating that BA alleviates LPS‐induced suppression of PPARγ expression and activation of the CD14 and NF‐κB pathways.

### 3.3 Impact of PPARγ Silencing on the Regulatory Effects of BA

**Figure 3:**
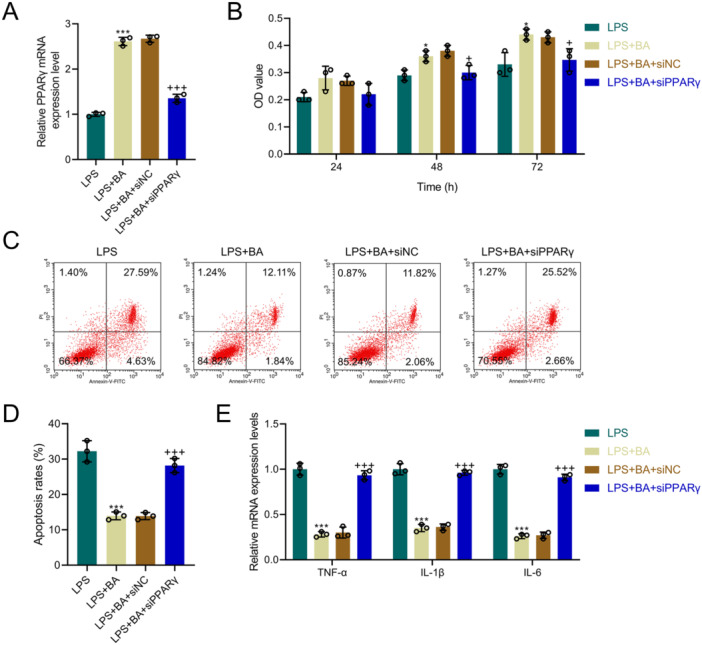
Silencing PPARγ reverses the protective effects of BA on cell viability, apoptosis, and inflammation in LPS‐treated AML12 cells. (A) QRT‐PCR analysis of PPARγ mRNA levels from the LPS, LPS + BA, LPS + BA + siNC, and LPS + BA + siPPARγ groups, with GAPDH as a loading control. (B) CCK8 assay results for cell viability at 24, 48, and 72 h from the LPS, LPS + BA, LPS + BA + siNC, and LPS + BA + siPPARγ groups. (C and D) Flow cytometry analysis of apoptosis rates from the LPS, LPS + BA, LPS + BA + siNC, and LPS + BA + siPPARγ groups. (E) QRT‐PCR analysis of TNF‐α, IL‐1β, and IL‐6 mRNA levels from the LPS, LPS + BA, LPS + BA + siNC, and LPS + BA + siPPARγ groups, with GAPDH as a loading control. *n* = 3. * versus LPS, *p* < 0.05, ***p < 0.001; ^+^ versus LPS + BA + siNC, *p* < 0.05, ^+++^ *p* < 0.001.

**Figure 4:**
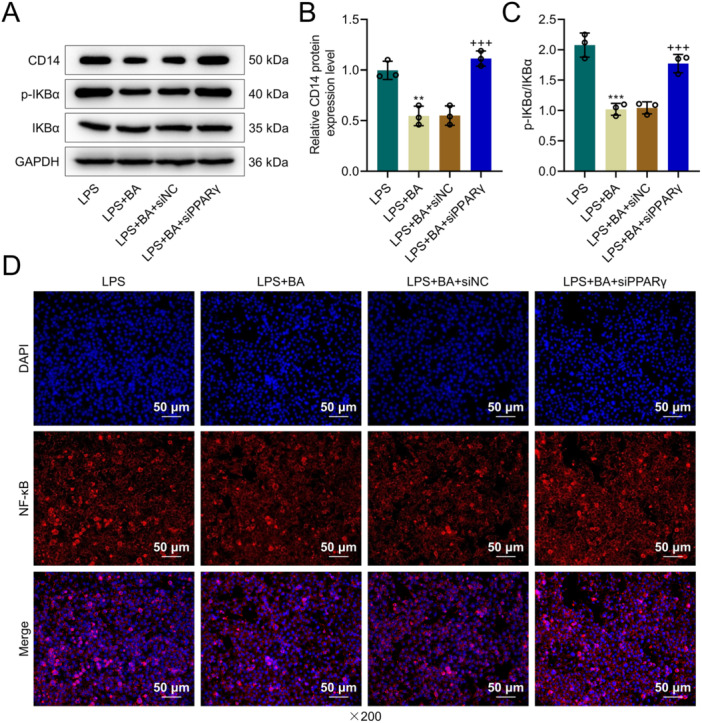
Silencing PPARγ reverses the inhibitory effects of BA on CD14 expression and NF‐κB pathway activation in LPS‐treated AML12 cells. (A–C) Western blot analysis of CD14, p‐IKBα, and IKBα protein levels in AML12 cells from the LPS, LPS + BA, LPS + BA + siNC, and LPS + BA + siPPARγ groups, with GAPDH as a loading control. (D) Immunofluorescence staining for NF‐κB (red) with nuclear counterstaining (blue) from the LPS, LPS + BA, LPS + BA + siNC, and LPS + BA + siPPARγ groups. Magnification = 200×, scale bar = 50 µm. *n* = 3. ** versus LPS, *p* < 0.01, ***p < 0.001; ^+++^ versus LPS + BA + siNC, *p* < 0.001.

QRT‐PCR results revealed that PPARγ levels were lower in the LPS + BA + siPPARγ group than LPS + BA + siNC group (*p* < 0.001, Figure 3A), proving successful transfection. CCK8 assays revealed that compared with the LPS + BA + siNC group, the OD values at 48 and 72 h were decreased in the LPS + BA + siPPARγ group (*p* < 0.05, Figure 3B), suggesting that silencing PPARγ could diminish BA‐promoted viability. The apoptosis rate was higher in the LPS + BA + siPPARγ group than the LPS + BA + siNC group (*p* < 0.001, Figure 3C,D), indicating that silencing PPARγ could reverse the apoptosis mitigation by BA. Further qRT‐PCR results demonstrated that levels of inflammatory cytokines TNF‐α, IL‐1β, and IL‐6 were significantly elevated in the LPS + BA + siPPARγ group compared to the LPS + BA + siNC group (*p* < 0.001, Figure 3E), suggesting that silencing PPARγ could reverse the mitigation of inflammatory markers by BA. Western blot results indicated that compared to the LPS + BA + siNC group, CD14 and p‐IKBα/IKBα proteins levels were elevated in the LPS + BA + siPPARγ group (*p* < 0.001, Figure 4A–C), indicating that silencing PPARγ can activate the NF‐κB pathway blocked by BA. Immunofluorescence analysis of nuclear translocation showed that the amount of nuclear translocation (red–blue overlap) was decreased in the LPS + BA group contrasted with the LPS group, and was increased in the LPS + BA + siPPARγ group contrasted with the LPS + BA + siNC group (Figure 4D), demonstrating that silencing PPARγ could activate NF‐κB nuclear translocation.

## 4 Discussion

Sepsis is a critical condition that often leads to organ failure and high mortality rates, with the liver being one of the most commonly affected organs. Given this impact, identifying effective therapeutic strategies to mitigate liver injury is paramount in the treatment of sepsis. BA, a flavonoid extracted from *S. baicalensis*, has various pharmacological activities, including antitumor, antimicrobial, and antioxidant, and it has the effect of improving liver damage [35, 36, 37]. BA upregulates macrophage TREM2 expression via TrKB–CREB1 pathway to attenuate acute inflammatory injury in acute‐on‐chronic liver failure; BA attenuates LPS/d‐Gal‐induced acute liver injury by blocking NLRP3 inflammasome [38]; BA pretreatment protects against liver ischemia/reperfusion injury via inhibition of autophagy in rats or inhibition of NF‐κB pathway in mice [39, 40]; BA protects against polymicrobial sepsis‐induced liver injury via inhibition of inflammation and apoptosis in mice [41]. This study also found that BA has the effect of improving liver damage, it offers significant protective benefits against liver damage caused by sepsis, mainly by regulating the PPARγ and CD14/NF‐κB pathways. Our findings provide a look at how BA enhances cell viability, decreases apoptosis, and modulates inflammatory responses in AML12 hepatocytes under septic conditions induced by LPS.

The efficacy of BA in improving cell viability and reducing apoptosis is particularly noteworthy. Our results showed that 25/100 µM BA notably mitigated the reduction in cell viability caused by LPS. Furthermore, BA treatment led to a significant decrease in apoptosis rates, highlighting its potential to protect liver cells during the acute phase of sepsis. These observations are consistent with previous studies that have identified BA's cytoprotective properties in various cellular models [24, 26]. For instance, BA protects renal cells from hypoxia‐induced apoptosis and cardiac cells from oxidative stress, underscoring its broad protective capabilities across different types of tissue and stress conditions [26].

The anti‐inflammatory effects of BA were also demonstrated by the significant downregulation of TNF‐α, IL‐1β, and IL‐6. These pro‐inflammatory cytokines play essential roles in the inflammatory reaction in sepsis and are often associated with the progression of organ dysfunction. By downregulating these cytokines, BA may help in curtailing the systemic inflammatory response that exacerbates liver damage during sepsis. The role of BA in modulating these cytokines further elucidates its potential active mechanism, which likely through dampening NF‐κB pathway, a key signaling cascade activated during sepsis.

Furthermore, our study emphasizes the influence of PPARγ in exerting a protective effects for BA. The silencing of PPARγ via siRNA not only reversed the protective effects of BA but also underscored the importance of this nuclear receptor in regulating inflammatory responses within the liver during septic challenges. PPARγ is known to exert anti‐inflammatory effect via repressing NF‐κB‐related factors, and its activation has been linked to beneficial outcomes in various inflammatory diseases [42]. The modulation of PPARγ by BA presents a novel therapeutic pathway that could be exploited further to develop treatments for sepsis and other inflammatory conditions.

The suppression of the CD14/NF‐κB pathway by BA provides another layer of understanding into its mechanism of action. The hallmark of sepsis is NF‐κB activation, which is vital for the expression of pro‐inflammatory genes [27]. By inhibiting this pathway, BA could potentially reduce the hepatic and systemic damage associated with sepsis‐induced inflammation. This finding aligns with previous research demonstrating CD14 as a co‐receptor in mediating LPS‐induced NF‐κB activation and suggests that targeting this pathway could yield substantial therapeutic benefits [27].

This study has confirmed that baicalin exerts a protective effect on sepsis‐induced liver injury through the PPARγ and CD14/NF‐κB signaling pathway. However, its clinical application still needs to take into account pharmacokinetic characteristics and safety factors. The pharmacokinetic characteristics of BA including gastrointestinal hydrolysis, enterohepatic circulation, carrier‐mediated transport, and complex metabolic processes [25, 37]. Moreover, the pharmacokinetic properties of BA will change under different pathological conditions. Due to the competition of metabolic enzymes and protein binding, BA may interact with other drugs in the combined therapy. It has been reported that BA affected the metabolism of other drugs by inhibiting CYP3A4 activity, and special caution should be exercised when used in combination with other drugs [43]. Current pharmacological and clinical studies have shown that BA is safe at therapeutic doses, there are mild adverse reactions [44, 45]. Future research should include more comprehensive toxicological evaluations and drug interaction studies to further promote the clinical application of baicalein in the treatment of sepsis‐related liver injury. In addition, the oral bioavailability of baicalin is relatively low. Future research should focus on the influence of pathological conditions on the pharmacokinetics of baicalin, and explore new drug delivery systems to enhance its bioavailability and targeting ability, thereby better exerting its therapeutic effect on liver injury caused by sepsis.

While our findings are promising, they are not without limitations. The use of a single cell line and the in vitro setting may not fully capture the complex interplay of cellular responses in a living organism during sepsis. Therefore, further studies involving animal models and eventually clinical trials are necessary to confirm the effectiveness and safety of BA for treating sepsis‐induced liver injury. Additionally, exploring the interactions of BA with other signaling pathways and its effects on other organs affected by sepsis would provide a more comprehensive view of its therapeutic potential.

In summary, this study offers compelling proof that BA not only preserves hepatocyte viability but also modulates crucial inflammatory pathways involved in sepsis. These findings suggest that BA, through its action on PPARγ and the CD14/NF‐κB pathway, holds promise as a therapeutic option for addressing liver damage caused by sepsis.

## Author Contributions

**Hui Wang:** conceptualization, methodology, investigation, formal analysis, writing – original draft, visualization. **Mengmeng Guo:** investigation, data curation, validation, writing – review and editing. **Xueling Zeng:** resources, software, validation, writing – review and editing. **Yuqi Hu:** investigation, data curation, formal analysis, writing – review and editing. **Jiawei Ma:** methodology, resources, supervision, writing – review and editing. **Yufei Chang:** conceptualization, funding acquisition, project administration, supervision, writing – review and editing, corresponding author responsibility.

## Ethics Statement

The authors have nothing to report.

## Conflicts of Interest

The authors declare no conflicts of interest.

## Supporting information

Supplemental Figure‐WB Original Figure.

## Data Availability

The analyzed data sets generated during the study are available from the corresponding author on reasonable request.
